# Supplementary material for: Fecal Pollution Drives Antibiotic Resistance and Class 1 Integron Abundance in Aquatic Environments of the Bolivian Andes Impacted by Mining and Wastewater
Source: Microorganisms. 2020 Jul 26;8(8):1122. doi: 10.3390/microorganisms8081122 (PMC7464395; doi:10.3390/microorganisms8081122)

**Figure S1.** **Plasmid-containing the construct with ARGs**. The cloning vector pUC57 **(A)** and the construct inserted into the vector **(B).** The construct including different ARGs, class 1 integrase (*int1*) and an indicator of human fecal bacteria (crAssphage) was inserted in the multi-cloning site (MCS) of the vector at the restriction site XbaI. The order of genes in the construct is shown in the map. The construct included ARGs for B-lactams (*acc-3, blaIMP-2, blaIMP-5, blaIMP-12 and blaOXA-2*), macrolides (*msrA*), methicillin (*mecA*), quinolones (*qnrB1, qnrB5* and *qnrS1*), sulfonamides (*sul1* and *sul2*) and tetracycline (*tetA* and *tetB*). Primers for the different genes included in the construct are described in Table1 of the manuscript. The map of the vector was obtained from SnapGene software (www.snapgene.com).


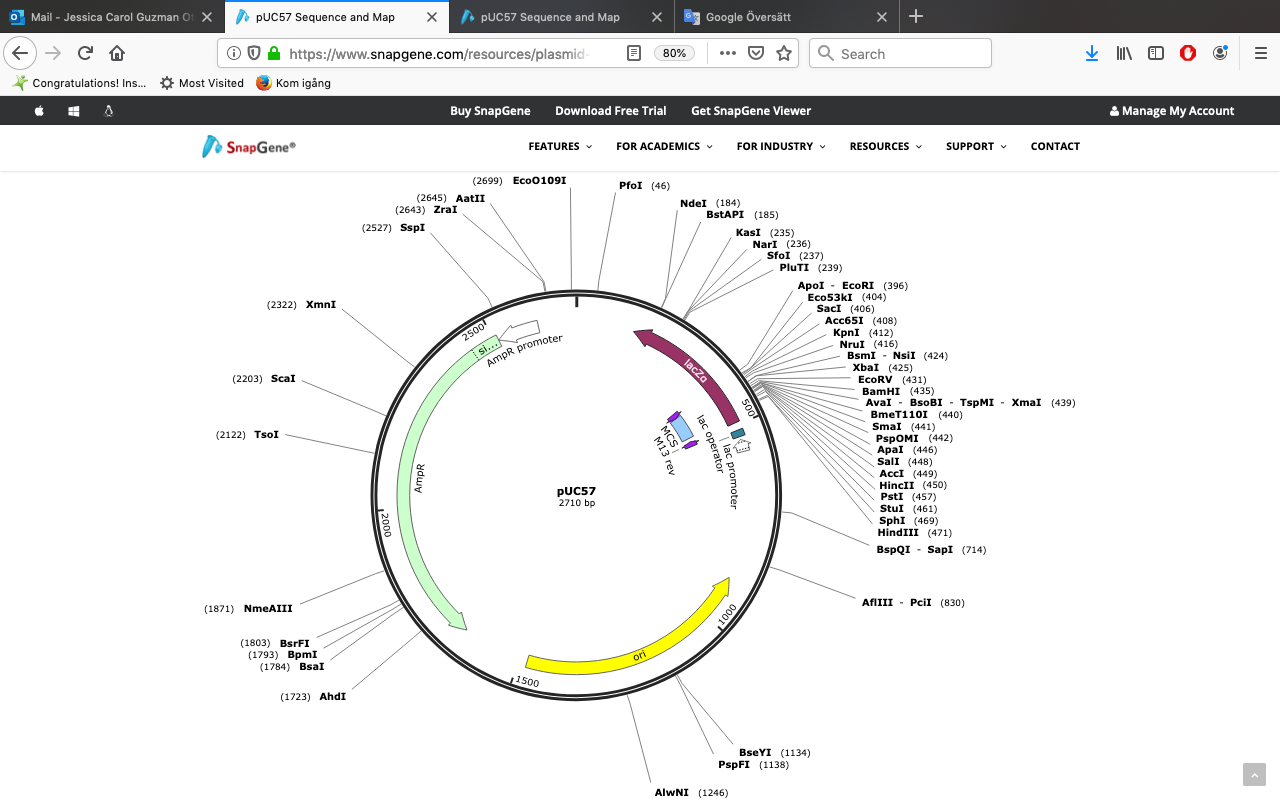


B.

A.


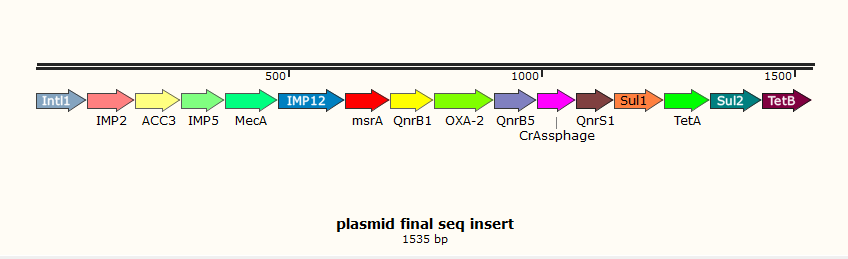

Supplement: Supplementary file 1 [file microorganisms-08-01122-s001.zip › S1 Fig.docx]
