# Supplementary material for: Fecal Pollution Drives Antibiotic Resistance and Class 1 Integron Abundance in Aquatic Environments of the Bolivian Andes Impacted by Mining and Wastewater
Source: Microorganisms. 2020 Jul 26;8(8):1122. doi: 10.3390/microorganisms8081122 (PMC7464395; doi:10.3390/microorganisms8081122)
Supplement: Supplementary file 1 [file microorganisms-08-01122-s001.zip › S1 table.docx]

**Table S1. List of ARG included in the qPCR array of Qiagen**.

| **Well** | **Species/gene** | **Antibiotic classification / virulence factor gene description** | **Also detects / associated species (virulence factor gene)** |
| --- | --- | --- | --- |
| A1 | AAC(6)-Ib-cr | Fluoroquinolone resistance |  |
| A2 | aacC1 | Aminoglycoside-resistance |  |
| A3 | aacC2 | Aminoglycoside-resistance |  |
| A4 | aacC4 | Aminoglycoside-resistance |  |
| A5 | aadA1 | Aminoglycoside-resistance |  |
| A6 | aphA6 | Aminoglycoside-resistance |  |
| A7 | BES-1 | Class A beta-lactamase |  |
| A8 | BIC-1 | Class A beta-lactamase |  |
| A9 | CTX-M-1 Group | Class A beta-lactamase | Detects CTX-M-1 type (37 variants) |
| A10 | CTX-M-8 Group | Class A beta-lactamase | Detects CTX-M-8 type (3 variants) |
| A11 | CTX-M-9 Group | Class A beta-lactamase | Detects CTX-M-9 type (40 variants) |
| A12 | GES | Class A beta-lactamase | GES,IBC |
| B1 | IMI & NMC-A | Class A beta-lactamase | NMC-A,IMI-2,IMI-3 |
| B2 | KPC | Class A beta-lactamase | KPC-1,KPC-2,KPC-3,KPC-4,KPC-5,KPC-6,KPC-7,KPC-8,KPC-9,KPC-10,KPC-11 |
| B3 | Per-1 group | Class A beta-lactamase | Per-1,Per-3,Per-4,Per-5 |
| B4 | Per-2 group | Class A beta-lactamase | Per-2,Per-6 |
| B5 | SFC-1 | Class A beta-lactamase |  |
| B6 | SFO-1 | Class A beta-lactamase |  |
| B7 | SHV | Class A beta-lactamase |  |
| B8 | SHV(156D) | Class A beta-lactamase |  |
| B9 | SHV(156G) | Class A beta-lactamase |  |
| B10 | SHV(238G240E) | Class A beta-lactamase |  |
| B11 | SHV(238G240K) | Class A beta-lactamase |  |
| B12 | SHV(238S240E) | Class A beta-lactamase |  |
| C1 | SHV(238S240K) | Class A beta-lactamase |  |
| C2 | SME | Class A beta-lactamase | SME-1,SME-2,SME-3 |
| C3 | TLA-1 | Class A beta-lactamase |  |
| C4 | VEB | Class A beta-lactamase | VEB-1,VEB-2,VEB-3,VEB-4,VEB-5,VEB-6,VEB-7 |
| C5 | ccrA | Class B beta-lactamase |  |
| C6 | IMP-1 group | Class B beta-lactamase | IMP-1,IMP-3,IMP-4,IMP-6,IMP-10,IMP-25,IMP-26 |
| C7 | IMP-12 group | Class B beta-lactamase | IMP-12,IMP-14,IMP-16,IMP-18 |
| C8 | IMP-2 group | Class B beta-lactamase | IMP-2,IMP-8,IMP-11,IMP-19,IMP-20,IMP-21,IMP-24 |
| C9 | IMP-5 group | Class B beta-lactamase | IMP-5,IMP-7,IMP-9,IMP-13,IMP-15,IMP-22 |
| C10 | NDM | Class B beta-lactamase | NDM-1,NDM-2 |
| C11 | VIM-1 group | Class B beta-lactamase | VIM-1,VIM-2,VIM-3,VIM-4,VIM-5,VIM-6,VIM-8,VIM-9,VIM-10,VIM-11,VIM-12,VIM-14,VIM-15,VIM-16,VIM-17,VIM-18,VIM-19,VIM-20,VIM-23,VIM-24,VIM-25,VIM-26 |
| C12 | VIM-13 | Class B beta-lactamase | VIM-13 (28 variants) |
| D1 | VIM-7 | Class B beta-lactamase |  |
| D2 | ACC-1 group | Class C beta-lactamase | ACC-1,ACC-2,ACC-4 |
| D3 | ACC-3 | Class C beta-lactamase |  |
| D4 | ACT 5/7 group | Class C beta-lactamase | ACT-5,ACT-7 |
| D5 | ACT-1 group | Class C beta-lactamase | ACT-1,ACT-2,ACT-3,ACT-4,ACT-6 |
| D6 | CFE-1 | Class C beta-lactamase |  |
| D7 | CMY-10 Group | Class C beta-lactamase | CMY-1,CMY-8,CMY-9,CMY-10,CMY-19 |
| D8 | DHA | Class C beta-lactamase | DHA-1,DHA-2,DHA-3,DHA-5,DHA-6,DHA-7 |
| D9 | FOX | Class C beta-lactamase | FOX-1,FOX-2,FOX-3,FOX-4,FOX-5,FOX-6,FOX-7 |
| D10 | LAT | Class C beta-lactamase | LAT-1,LAT-3,LAT-4,CMY-2 group |
| D11 | MIR | Class C beta-lactamase | MIR-1,MIR-2,MIR-3,MIR-4,MIR-5 |
| D12 | MOX | Class C beta-lactamase | MOX-1,MOX-2,MOX-3,MOX-4,MOX-5,MOX-6,MOX-7 |
| E1 | OXA-10 Group | Class D beta-lactamase | OXA-10,OXA-11,OXA-14,OXA-16,OXA-17,OXA-19,OXA-28,OXA-35,OXA-142,OXA-145,OXA-147 |
| E2 | OXA-18 | Class D beta-lactamase |  |
| E3 | OXA-2 Group | Class D beta-lactamase | OXA-2,OXA-15,OXA-32,OXA-34,OXA-141,OXA-161 |
| E4 | OXA-23 Group | Class D beta-lactamase | OXA-23,OXA-27,OXA-49,OXA-73,OXA-133,OXA-146,OXA-165,OXA-166,OXA-167,OXA-168,OXA-169,OXA-170,OXA-171 |
| E5 | OXA-24 Group | Class D beta-lactamase | OXA-24,OXA-25,OXA-26,OXA-40,OXA-72,OXA-139,OXA-160 |
| E6 | OXA-45 | Class D beta-lactamase |  |
| E7 | OXA-48 Group | Class D beta-lactamase | OXA-48,OXA-162,OXA-163,OXA-181 |
| E8 | OXA-50 Group | Class D beta-lactamase | OXA-50 group (50 variants) |
| E9 | OXA-51 Group | Class D beta-lactamase | OXA-51 group (65 variants) |
| E10 | OXA-54 | Class D beta-lactamase |  |
| E11 | OXA-55 | Class D beta-lactamase | OXA-55,OXA-SH |
| E12 | OXA-58 Group | Class D beta-lactamase | OXA-58,OXA-96,OXA-97,OXA-164 |
| F1 | OXA-60 | Class D beta-lactamase | OXA-60,OXA-60a,OXA-60b,OXA-60c |
| F2 | ereB | Erythromycin resistance |  |
| F3 | QepA | Fluoroquinolone resistance | QepA1,QepA2 |
| F4 | QnrA | Fluoroquinolone resistance | QnrA1,QnrA2,QnrA3,QnrA4,QnrA5,QnrA6,QnrA7 |
| F5 | QnrB-1 group | Fluoroquinolone resistance | QnrB1,QnrB2,QnrB3,QnrB6,QnrB7,QnrB9,QnrB13,QnrB14,QnrB15,QnrB16,QnrB17,QnrB18,QnrB20,QnrB23,QnrB24,QnrB29,QnrB30 |
| F6 | QnrB-31 group | Fluoroquinolone resistance | QnrB31,QnrB32 |
| F7 | QnrB-4 group | Fluoroquinolone resistance | QnrB4,QnrB11,QnrB12,QnrB22 |
| F8 | QnrB-5 group | Fluoroquinolone resistance | QnrB5,QnrB10,QnrB19 |
| F9 | QnrB-8 group | Fluoroquinolone resistance | QnrB8,QnrB21,QnrB25,QnrB27,QnrB28 |
| F10 | QnrC | Fluoroquinolone resistance |  |
| F11 | QnrD | Fluoroquinolone resistance |  |
| F12 | QnrS | Fluoroquinolone resistance | QnrS1,QnrS2,QnrS3,QnrS4 |
| G1 | ermA | Macrolide Lincosamide Streptogramin_b |  |
| G2 | ermB | Macrolide Lincosamide Streptogramin_b |  |
| G3 | ermC | Macrolide Lincosamide Streptogramin_b |  |
| G4 | mefA | Macrolide Lincosamide Streptogramin_b |  |
| G5 | msrA | Macrolide Lincosamide Streptogramin_b |  |
| G6 | oprj | Multidrug resistance efflux pump |  |
| G7 | oprm | Multidrug resistance efflux pump |  |
| G8 | tetA | Tetracycline efflux pump |  |
| G9 | tetB | Tetracycline efflux pump |  |
| G10 | vanB | Vancomycin resistance |  |
| G11 | vanC | Vancomycin resistance |  |
| H1 | mecA | Beta-lactam resistance |  |
